# Prognostic value of the S100B protein in newly diagnosed and recurrent glioma patients: a serial analysis

**DOI:** 10.1007/s11060-016-2204-z

**Published:** 2016-07-11

**Authors:** F. K. Holla, T. J. Postma, M. A. Blankenstein, T. J. M. van Mierlo, M. J. Vos, E. M. Sizoo, M. de Groot, B. M. J. Uitdehaag, J. Buter, M. Klein, J. C. Reijneveld, J. J. Heimans

**Affiliations:** 1 Department of Neurology, VU University Medical Center, PO Box 7057, 1007 MB Amsterdam, The Netherlands; 2 Clinical Chemistry, VU University Medical Center, Amsterdam, The Netherlands; 3 Department of Neurology, Medical Center Haaglanden, The Hague, The Netherlands; 4 Epidemiology and Biostatistics, VU University Medical Center, Amsterdam, The Netherlands; 5 Medical Oncology, VU University Medical Center, Amsterdam, The Netherlands; 6 Medical Psychology, VU University Medical Center, Amsterdam, The Netherlands

**Keywords:** Glioma, S100B, Survival, Chemotherapy, Prognosis

## Abstract

The S100B protein is associated with brain damage and a breached blood–brain barrier. A previous pilot study showed that high serum levels of S100B are associated with shorter survival in glioma patients. The aim of our study was to assess the prognostic value in terms of survival and longitudinal dynamics of serum S100B for patients with newly diagnosed and recurrent glioma. We obtained blood samples from patients with newly diagnosed and recurrent glioma before the start (baseline) and at fixed time-points during temozolomide chemotherapy. S100B-data were dichotomized according to the upper limit of the reference value of 0.1 μg/L. Overall survival (OS) was estimated with Kaplan–Meier curves and groups were compared with the log rank analysis. To correct for potential confounders a Cox regression analysis was used. We included 86 patients with newly-diagnosed and 27 patients with recurrent glioma. Most patients in both groups had baseline serum levels within normal limits. In the newly diagnosed patients we found no significant difference in OS between the group of patients with S100B levels >0.1 μg/L at baseline compared to those with <0.1 μg/L. In the patients with recurrent glioma we found a significantly shorter OS for patients with raised levels. In both groups, S100B values did not change significantly throughout the course of the disease. Serum S100B levels do not seem to have prognostic value in newly diagnosed glioma patients. In recurrent glioma patients S100B might be of value in terms of prognostication of survival.

## Introduction

Gliomas are the most common primary brain tumors in adults with a histological grade that ranges from low (WHO I, II) to high grade (WHO III, IV) [1]. Despite different treatment regimens, such as radio- and chemotherapy, which have evolved over the past decade, survival is still limited. Various prognostic factors have been identified, such as age, histology, extent of resection, corticosteroid use, and Karnofsky Performance Status (KPS) [2–7]. Currently the role of the genetic profile of gliomas is extensively being investigated and seems to be a strong prognostic factor in survival and treatment response in glioma patients [8–10]. However, an easy to use serum biomarker to predict the prognosis in glioma patients would still be an asset for patient care. Serum levels of the S100B protein may be valuable in this respect.

The S100B protein is one of the most studied markers of central nervous system (CNS) pathology. This cytoplasmic Ca^2+^-binding protein is highly expressed in the CNS where it is primarily secreted by astrocytes and other glial cell types such as Schwann cells and oligodendrocytes [11–13]. This protein is also widely distributed in tissues outside the nervous system, such as melanocytes, chondrocytes, skeletal muscle cells and adipose tissue [14–17].

Astrocytes leak and actively secrete S100B into the extracellular environment during metabolic stress, leading to raised levels in the cerebrospinal fluid (CSF) [18]. When the blood–brain barrier (BBB) is breached, the protein is subsequently released into the serum. Serum S100B is a suggested marker for BBB integrity, because levels are also raised after chemically opening the BBB in the absence of cerebral damage [19].

In clinical practice raised serum levels of S100B have been found in numerous neurological disorders, such as traumatic brain injury and acute stroke. In these conditions raised serum S100B levels are positively correlated with unfavourable neurological outcome, mortality and imaging characteristics [20–29]. Furthermore, serum S100B has an established role in the management of melanoma, especially in the prediction of response to systemic therapy [30–33]. Raised serum levels of the protein have a potential role in the prediction and detection of brain metastasis in lung cancer patients as well [34].

In glioma patients little is known about the prognostic value of serum S100B and its longitudinal behaviour during the course of the disease. Raised serum levels have been documented in glioblastoma patients [35]. Furthermore, a former pilot study showed a correlation between high serum S100B concentrations and a shorter survival in a small population of glioma patients [36]. Based on these results, we hypothesize that serum S100B can be helpful in predicting prognosis in glioma patients.

The aim of this study was to prospectively evaluate the prognostic value and the longitudinal dynamics in terms of overall survival (OS) of serum S100B in patients with newly diagnosed and recurrent glioma during treatment with chemotherapy.

## Patients and methods

Between September 2004 and January 2013 patients with histological proven, newly diagnosed or recurrent glioma who were scheduled for chemotherapy treatment and who visited the Neurology out-patient clinic of our institution, were included into a prospective study on the evaluation of side effects of chemotherapy. Informed consent was obtained by the treating physician and the protocol of this study was approved by the local Ethics Committee.

### Newly diagnosed glioma

Patients in this group were eligible if they were diagnosed with histologically proven glioma, had a good performance status, and were scheduled for postoperative treatment with concurrent radiotherapy (60 Gy in 30 fractions of 2 Gy) and chemotherapy (temozolomide 75 mg/m², 5 days/week for 6 weeks during radiotherapy) followed by six adjuvant courses of chemotherapy in the form of oral temozolomide 150–200 mg/m² in cycles of 5 days in 4 weeks [37]. Serum samples were obtained at baseline (before the concomitant phase), before the adjuvant phase, and every three cycles thereafter until progressive disease or chemotherapy-induced toxicity was apparent. From patients that had completed six adjuvant courses without radiological and/or clinical progression, serum samples were also acquired at 3 and 7 months after treatment.

### Recurrent glioma

For this group, patients were included who were diagnosed with radiologically and/or surgically confirmed tumor recurrence of a previously histologically confirmed glioma, and who were scheduled for treatment with chemotherapy.

Chemotherapy consisted of oral temozolomide in two possible regimens: a short cycle of 5 consecutive days in 4 weeks in doses of 150–200 mg/m², or a long cycle of 21 consecutive days in 4 weeks (‘3 weeks on, 1 week off’) in doses of 75 mg/m².

If patients previously had been treated with temozolomide, they received PCV: a combination chemotherapy of 110 mg/m² oral lomustine (CCNU) on day 1, followed by 1.4 mg/m² intravenous vincristine on day 8, and 60 mg/m² of oral procarbazine from day 8 until 23 with repeated administration of 1.4 mg/m² intravenous vincristine on day 29. Cycles were repeated until clinical and/or radiological progression or chemotherapy-induced toxicity was apparent.

Serum samples in this group were obtained at baseline (before start of first cycle) and every three cycles thereafter during chemotherapy treatment.

### Chemical analysis

All patients were seen in a single clinic. Blood was collected at the physician’s office by venous puncture and transported immediately to the adjacent clinical chemistry laboratory where it was centrifuged, aliquoted, frozen and stored at −20 °C until assay. The entire procedure was completed within 2 h from phlebotomy.

Serum S100B was measured by the commercially available immunologic assay Elecsys S100 (Roche/Cobas®) with a detection limit of <0.005 μg/L. Intra-assay variation at 0.06 and 0.31 µg/L was 1.9 and 1.0 % respectively (n = 10). Inter-assay variation at 0.197 and 2.46 µg/L was 1.6 and 5.6 % respectively (n = 16; three lots of controls). All samples were analyzed without knowledge of the clinical status of the patient. Data were dichotomized with a cut off value of 0.1 µg/L, which is considered to be the estimated upper limit of the reference range in serum [38–40].

### Definition of outcome variables

Patients’ clinical records were reviewed to obtain information concerning clinical and survival data. OS was defined as the interval between the date of baseline blood sampling and date of death. Tumor progression was defined according to the RANO criteria [41]. Age, tumor grade, baseline KPS and corticosteroid use were included as co-variables.

### Statistical analysis

Kaplan–Meier curves were used to estimate OS times. To calculate significance between survival curves a log-rank test was used. A Cox proportional hazard analysis was used to assess the prognostic value of serum S100B, corrected for potential confounders. To calculate a significant change of S100B value during follow up a Mann–Whitney *U* test was used. A *p*-value of <0.05 was used to determine significance. The analyses were performed with SPSS software (version 20.0).

## Results

### Patient characteristics

Tables 1 and 2 list the patient characteristics for the newly diagnosed and recurrent glioma patient groups respectively.

**Table 1:** Patient characteristics newly diagnosed glioma group

Characteristic	Number of patients (n = 86)
Male/female ratio	59/27
Median age (y; range)^a^	56 (18–78)
Baseline corticosteroids yes/no^b^	23/63
Median baseline KPS (range)	90 (50–100)
Histology	
GBM	85
O/A III	1
Type of surgery	
Resection	78
Stereotactic biopsy	8
Median total dose rth (Gy)	60 (42–60)
Median number TMZ courses (n)^d^	6 (0–13)
Median time primary diagnosis—baseline S100B (days)	18 (6–56)

*KPS* Karnofsky Performance Score, *rth* radiotherapy, *TMZ* temozolomide, *GBM* glioblastoma, *O*/*A* oligo-astrocytoma

^a^Age at primary diagnosis

^b^Corticosteroid = oral dexamethason

^c^Type of surgery at primary diagnosis

^d^Adjuvant TMZ courses

**Table 2:** Patient characteristics recurrent glioma group

Characteristic	Number of patients (n = 27)
Male/female ratio	17/10
Median age (y; range)^a^	47 (22–66)
Baseline corticosteroids yes/no^b^	13/14
Median baseline KPS (range)	80 (50–100)
Histology	
A II–A III–O II–O III–O IV^c^–O/A II–O/A III–GBM	3–4–2–3–1–1–2–11
Type of chemotherapy received	
TMZ 5/28/TMZ 3 weeks on, 1 week off/PCV	22/4/1
Median number chemotherapy courses (n; range)	6 (1–18)
Median time primary diagnosis—baseline S100B (mo, range)	26 (1–223)

*KPS* Karnofsky Performance Score, *rth* radiotherapy, *TMZ* temozolomide, *PCV* procarbazine, CCNU and vincristine, *A* astrocytoma, *O* oligodendroglioma, *O*/*A* oligo-astrocytoma, *GBM* glioblastoma

^a^Age at inclusion date

^b^Corticosteroid = oral dexamethason

^c^Glioblastoma with oligodendroglial component

Ninety-seven patients were newly diagnosed with glioma, had undergone cranial surgery and were scheduled for postoperative chemo-radiation therapy. In 11 patients a baseline blood sample could not be obtained and were therefore excluded from further analysis, resulting in 86 included patients. The median OS since baseline blood sampling was 14 months (range 2–60). As of August 2013, 25 out of 86 patients had not died and were thus censored to this date.

Twenty-seven patients were diagnosed with recurrent glioma according to the RANO criteria and were scheduled for treatment with chemotherapy. Tumor recurrence was confirmed by MR imaging in 15 patients, and histological confirmed in 12 patients. Twenty patients were treated for their first recurrence and seven patients for their second. All patients had previously undergone surgical resection or stereotactic biopsy, of which six patients were re-operated once and five patients twice. Twenty patients received radiotherapy in an earlier stage of disease and five patients were already treated with chemotherapy of which one patient was treated for another malignancy (sigmoid carcinoma).

The median survival after baseline blood sampling was 12 months (1–74) with 4 out of 27 patients censored.

### Serum S100B measurements

**Fig. 1:**
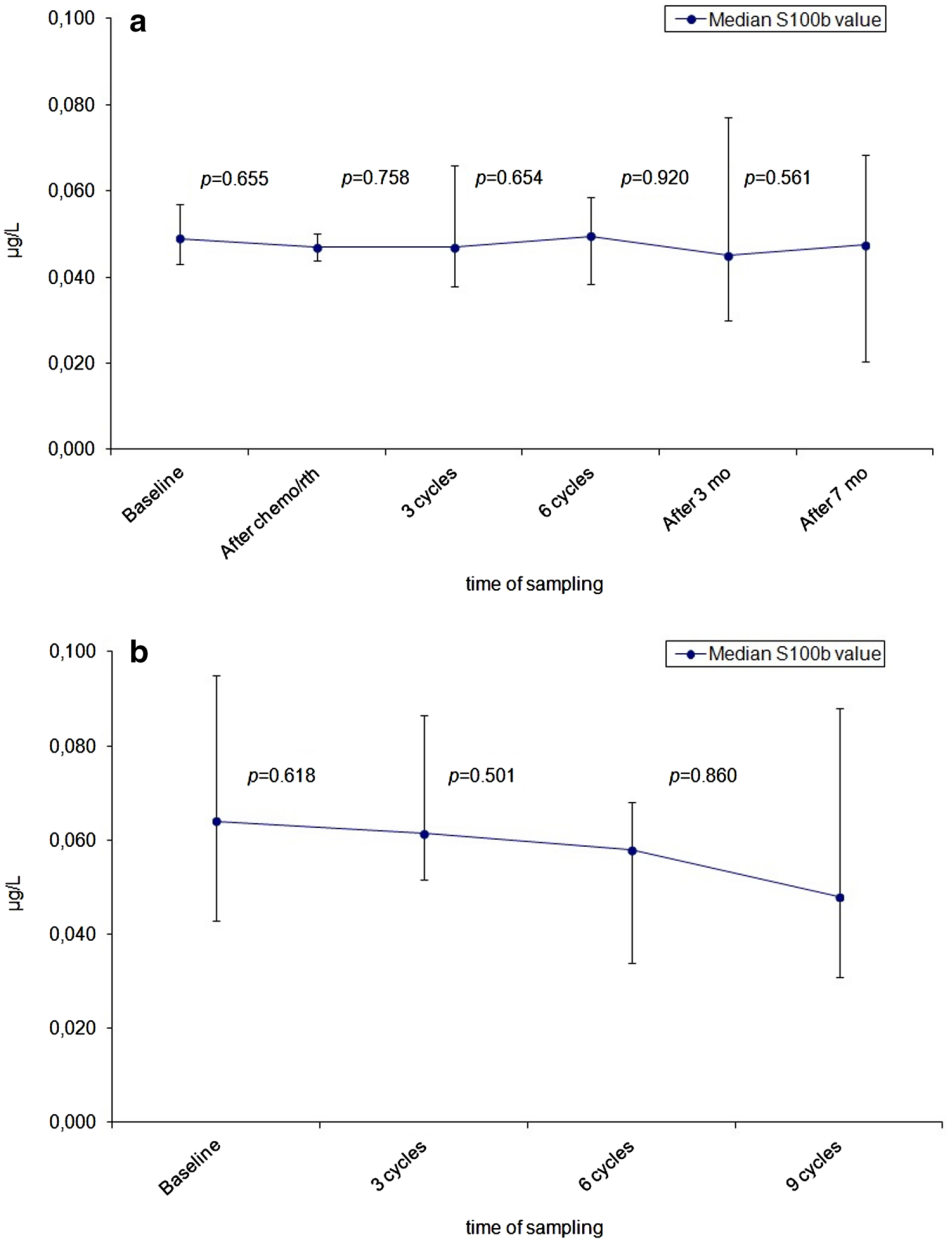
Longitudinal dynamics for median serum S100B values during the course of therapy for **a** newly diagnosed glioma and **b** recurrent glioma. *Error bars* represent 95 % confidence intervals

In the newly diagnosed group a median number of three serum samples (range 1–6) was obtained per patient. The median baseline serum S100B value was 0.049 µg/L (range 0.015–0.459). Seven patients (8 %) had levels above the upper limit of the reference range of 0.1 µg/L, all diagnosed with glioblastoma multiforme with two patients demonstrating remarkably high serum levels of 0.313 and 0.459 µg/L. Median serum levels did not change significantly during follow-up (Fig. 1a). Age did not seem to interfere with S100B values as well (p = 0.557, independent *t* test).

The median number of serum samples obtained in the recurrent group was 2 (range 1–7) and the median baseline S100B value was 0.064 µg/L (0.020–0.430). Seven patients (26 %) had levels exceeding the reference value, of which six had been diagnosed with glioblastoma multiforme and one patient with oligodendroglioma WHO III. There was one patient with a high serum level of 0.430 µg/L, who had been diagnosed with glioblastoma multiforme. As in the newly diagnosed group no significant changes were found in median serum S100b levels during treatment with chemotherapy (Fig. 1b). There was no significant difference in age between these two groups (p = 0.831, independent *t* test).

### Survival analysis

In the newly diagnosed group one of the two patients demonstrating the highest serum levels showed a relatively short survival (8 months) since baseline blood sampling. However, the other patient was still alive as of August 2013 with a survival of 23 months from baseline blood sampling.

The patient demonstrating the highest serum level in the recurrent group also showed a relatively short survival compared to the median survival since baseline blood sampling (6 vs. 12 months, respectively). Only one patient demonstrated a shorter survival of 1 month (baseline serum S100B: 0.107 µg/L).

When patients in both groups were dichotomized by the estimated upper limit of the reference range of 0.1 µg/L, no significant difference in survival was found in the newly diagnosed group.

**Fig. 2:**
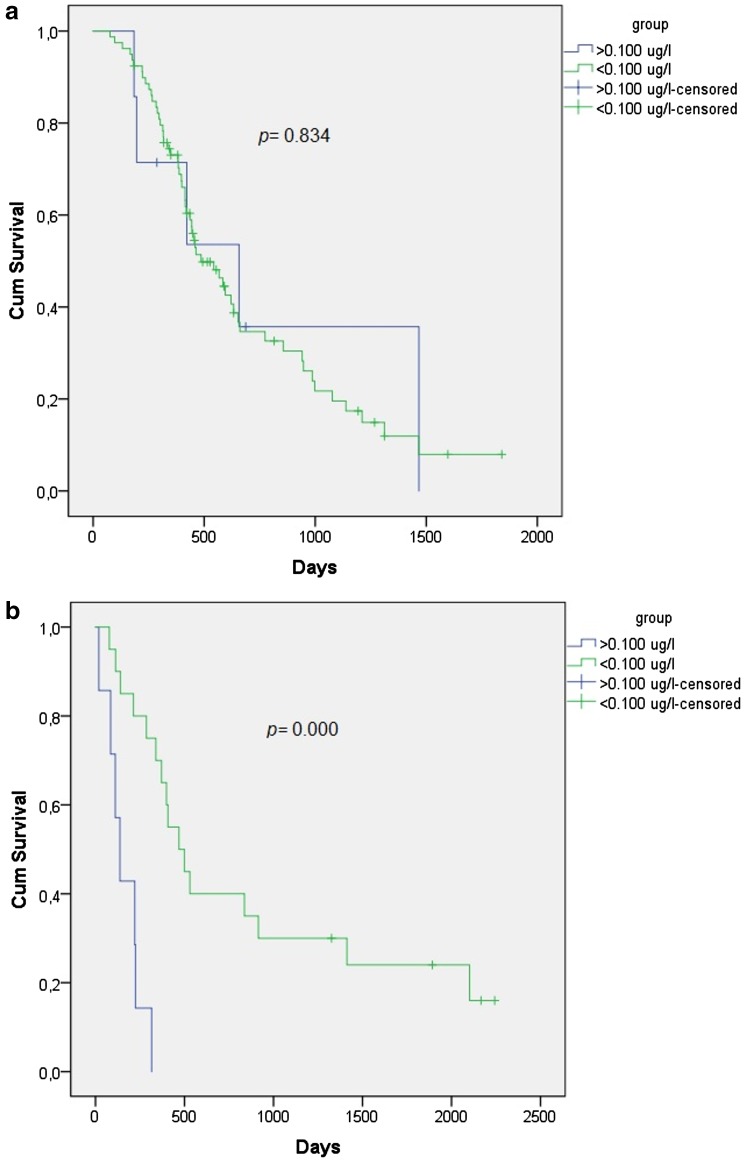
Kaplan–Meier curves for overall survival dichotomized according to the upper limit of the reference range of 0.100 µg/L for **a** newly diagnosed glioma and **b** recurrent glioma

In the recurrent group a significantly shorter survival of median 4 months (range 1–10) was found for patients (n = 7) with serum levels >0.1 µg/L compared to 16 months median survival (range 3–69) for patients (n = 20) with serum levels <0.1 µg/L (log rank-test, p = 0.000, Fig. 2). This remained significant after correction for age, tumor grade (dichotomized according to WHO high grade vs. low grade), KPS at baseline and corticosteroid use at baseline with Cox proportional hazard analysis (hazard ratio 4.1, 95 % confidence interval 1.1–15.2).

## Discussion

In the current study we found that the majority of newly diagnosed and recurrent glioma patients have serum S100B levels within normal limits. The median serum levels at baseline for the newly diagnosed and recurrent groups were respectively 0.049 and 0.064 µg/L. These findings are fairly in line with other studies, which also found median values within the reference range in glioma patients at different stages of disease [36, 42–44]. Furthermore, no significant change over the course of treatment was found in both patient groups.

Although a significantly shorter survival was found for patients with raised levels in the recurrent glioma group, this was not found for the, much larger, newly diagnosed glioma group.

Regarding the correlation of serum S100B levels and survival in glioma patients few data are available in literature. Mutlu et al. found no significant association between pre-treatment serum S100B values and OS in patients (n = 27), who were recently diagnosed with glioblastoma [43]. These results are confirmed by our findings in the newly diagnosed glioma group. Vos et al. found an association between serum levels above 0.09 µg/L and a shorter survival in glioma patients at different stages of treatment and disease (n = 20) [36]. These results are in line with the findings in our recurrent group. We have no clear explanation for this phenomenon. However, the number of patients in both the recurrent group in our study and the group investigated by Vos et al. is small and in various stages of disease, and should thus be interpreted with caution.

Several hypotheses concerning raised serum S100B levels in glioma patients have been postulated:

(1) proliferating brain tumors can cause damage to astrocytes by causing a disruption of cell integrity, leading to an increased release of S100B, (2) disruption of the BBB also leads to an increase in serum S100B levels, even in absence of cerebral damage [19, 45, 46]. (3) craniotomy itsels may lead to higher S100B levels [47].

Marchi et al. published a mathematical model in which it is postulated that maximum serum levels achievable after BBB-failure are around 0.34 µg/L and thus, levels exceeding these values are probably due to other factors such as non-CNS release or concomitant brain damage [46].

Breaching of the BBB can be assessed by the appearance of contrast enhancement on MRI, which is especially apparent in high grade gliomas. All patients in our study showed contrast enhancement on MRI, indicating breaching of BBB. According to the model of Marchi et al. it would seem logical that the majority of patients would have elevated serum S100B values, however most patients showed serum levels within normal limits throughout various stages of disease. Furthermore, no clear correlation (data not shown) could be found between the degree of contrast enhancement and serum levels. In contrast, brain damage and disruption of the BBB caused by traumatic brain injury and acute stroke does seem to lead to raised serum S100B levels which is correlated to clinical outcome and mortality [22, 25, 29]. It remains unclear why glioma patients do not show significant elevation of serum levels.

Syeda et al. describe temporarily (<7 days) high serum levels of S100B in patients who underwent craniotomy for a brain tumor, suggesting that these values are probably due to surgically induced tissue damage [47]. In our study the median interval between baseline blood sampling and last surgery was 18 and 247 days, in newly diagnosed and recurrent glioma patients, respectively, which is significantly longer than the interval described in literature. Furthermore, we found no consistent trend in S100B levels in patients with resection vs. biopsy in this study.

There are several limitations to this study. First, blood sampling was ceased when clinical and/or radiological progression was apparent. As such, it was not possible to study the values of serum S100B after tumor progression. Therefore, it is recommended that serum measurements are continued in patients that show clinical and/or radiological progression and subsequently switch to alternative treatments. Second, patients in the recurrent group showed heterogeneity regarding the stages of disease and previous treatment, which may have influenced survival data and/or serum S100B levels. Third, the genetic profile of our glioma patients was not assessed as a confounding factor in this study, which may have biased the results concerning the prognostic value of serum S100B. Last, as mentioned earlier, the number of patients in the recurrent group was low (n = 27), which may have led to biased results.

In conclusion, the majority of glioma patients have normal serum S100B values which remains within the normal limits throughout the course of the disease. Serum S100B does not seem to have prognostic value in newly diagnosed glioma patients. In recurrent glioma patients S100B might be of value in terms of prognostication of survival.

## References

[1] Louis DN, Ohgaki H, Wiestler OD, Cavenee WK, Burger PC, Jouvet A (2007). The 2007 WHO classification of tumours of the central nervous system. Acta Neuropathol.

[2] Nuno M, Birch K, Mukherjee D, Sarmiento JM, Black KL, Patil CG (2013). Survival and prognostic factors of anaplastic gliomas. Neurosurgery.

[3] Sanai N, Berger MS (2008). Glioma extent of resection and its impact on patient outcome. Neurosurgery.

[4] Back MF, Ang ELL, Ng WH, See SJ, Lim CCT, Chan SP (2007). Improved median survival for glioblastoma multiforme following introduction of adjuvant temozolomide chemotherapy. Ann Acad Med Singap.

[5] Donato V, Papaleo A, Castrichino A, Banelli E, Giangaspero F, Salvati M (2007). Prognostic implication of clinical and pathologic features in patients with glioblastoma multiforme treated with concomitant radiation plus temozolomide. Tumori.

[6] Curran WJJ, Scott CB, Horton J, Nelson JS, Weinstein AS, Fischbach AJ (1993). Recursive partitioning analysis of prognostic factors in three Radiation Therapy Oncology Group malignant glioma trials. J Natl Cancer Inst.

[7] Cairncross G, Berkey B, Shaw E, Jenkins R, Scheithauer B, Brachman D (2006). Phase III trial of chemotherapy plus radiotherapy compared with radiotherapy alone for pure and mixed anaplastic oligodendroglioma: Intergroup Radiation Therapy Oncology Group Trial 9402. J Clin Oncol.

[8] Hegi ME, Diserens AC, Gorlia T, Hamou MF, de Tribolet N, Weller M (2005). MGMT gene silencing and benefit from temozolomide in glioblastoma. N Engl J Med.

[9] Cohen AL, Colman H (2015). Glioma biology and molecular markers. Cancer Treat Res.

[10] Speirs CK, Simpson JR, Robinson CG, DeWees TA, Tran DD, Linette G (2015). Impact of 1p/19q codeletion and histology on outcomes of anaplastic gliomas treated with radiation therapy and temozolomide. Int J Radiat Oncol Biol Phys.

[11] Ludwin SK, Kosek JC, Eng LF (1976). The topographical distribution of S-100 and GFA proteins in the adult rat brain: an immunohistochemical study using horseradish peroxidase-labelled antibodies. J Comp Neurol.

[12] Brockes JP, Fields KL, Raff MC (1979). Studies on cultured rat Schwann cells. I. Establishment of purified populations from cultures of peripheral nerve. Brain Res.

[13] Rickmann M, Wolff JR (1995). S100 protein expression in subpopulations of neurons of rat brain. Neuroscience.

[14] Cocchia D, Michetti F, Donato R (1981). Immunochemical and immuno-cytochemical localization of S-100 antigen in normal human skin. Nature.

[15] Stefansson K, Wollmann RL, Moore BW, Arnason BG (1982). S-100 protein in human chondrocytes. Nature.

[16] Tubaro C, Arcuri C, Giambanco I, Donato R (2010). S100B protein in myoblasts modulates myogenic differentiation via NF-kappaB-dependent inhibition of MyoD expression. J Cell Physiol.

[17] Michetti F, Dell’Anna E, Tiberio G, Cocchia D (1983). Immunochemical and immunocytochemical study of S-100 protein in rat adipocytes. Brain Res.

[18] Gerlach R, Demel G, Konig HG, Gross U, Prehn JHM, Raabe A (2006). Active secretion of S100B from astrocytes during metabolic stress. Neuroscience.

[19] Kapural M, Krizanac-Bengez L, Barnett G, Perl J, Masaryk T, Apollo D (2002). Serum S-100beta as a possible marker of blood–brain barrier disruption. Brain Res.

[20] Abraha HD, Butterworth RJ, Bath PM, Wassif WS, Garthwaite J, Sherwood RA (1997). Serum S-100 protein, relationship to clinical outcome in acute stroke. Ann Clin Biochem.

[21] Sanchez-Pena P, Pereira AR, Sourour NA, Biondi A, Lejean L, Colonne C (2008). S100B as an additional prognostic marker in subarachnoid aneurysmal hemorrhage. Crit Care Med.

[22] Mercier E, Boutin A, Lauzier F, Fergusson DA, Simard JF, Zarychanski R (2013). Predictive value of S-100beta protein for prognosis in patients with moderate and severe traumatic brain injury: systematic review and meta-analysis. BMJ.

[23] Wiesmann M, Steinmeier E, Magerkurth O, Linn J, Gottmann D, Missler U (2010). Outcome prediction in traumatic brain injury: comparison of neurological status, CT findings, and blood levels of S100B and GFAP. Acta Neurol Scand.

[24] Gradisek P, Osredkar J, Korsic M, Kremzar B (2012). Multiple indicators model of long-term mortality in traumatic brain injury. Brain Inj.

[25] Foerch C, Singer OC, Neumann-Haefelin T, du Mesnil de Rochemont R, Steinmetz H, Sitzer M (2005). Evaluation of serum S100B as a surrogate marker for long-term outcome and infarct volume in acute middle cerebral artery infarction. Arch Neurol.

[26] Sienkiewicz-Jarosz H, Galecka-Wolska M, Bidzinski A, Turzynska D, Sobolewska A, Lipska B (2009). Predictive value of selected biochemical markers of brain damage for functional outcome in ischaemic stroke patients. Neurol Neurochir Pol.

[27] Moritz S, Warnat J, Bele S, Graf BM, Woertgen C (2010). The prognostic value of NSE and S100B from serum and cerebrospinal fluid in patients with spontaneous subarachnoid hemorrhage. J Neurosurg Anesthesiol.

[28] Gonzalez-Garcia S, Gonzalez-Quevedo A, Fernandez-Concepcion O, Pena-Sanchez M, Menendez-Sainz C, Hernandez-Diaz Z (2012). Short-term prognostic value of serum neuron specific enolase and S100B in acute stroke patients. Clin Biochem.

[29] Dassan P, Keir G, Brown MM (2009). Criteria for a clinically informative serum biomarker in acute ischaemic stroke: a review of S100B. Cerebrovasc Dis.

[30] Martenson ED, Hansson LO, Nilsson B, von Schoultz E, Mansson Brahme E, Ringborg U (2001). Serum S-100b protein as a prognostic marker in malignant cutaneous melanoma. J Clin Oncol.

[31] Kruijff S, Bastiaannet E, Brouwers AH, Nagengast WB, Speijers MJ, Suurmeijer AJH (2012). Use of S-100B to evaluate therapy effects during bevacizumab induction treatment in AJCC stage III melanoma. Ann Surg Oncol.

[32] Hamberg AP, Korse CM, Bonfrer JM, de Gast GC (2003). Serum S100B is suitable for prediction and monitoring of response to chemoimmunotherapy in metastatic malignant melanoma. Melanoma Res.

[33] Kluger HM, Hoyt K, Bacchiocchi A, Mayer T, Kirsch J, Kluger Y (2011). Plasma markers for identifying patients with metastatic melanoma. Clin Cancer Res.

[34] Vogelbaum MA, Masaryk T, Mazzone P, Mekhail T, Fazio V, McCartney S (2005). S100beta as a predictor of brain metastases: brain versus cerebrovascular damage. Cancer.

[35] Lyubimova NV, Toms MG, Popova EE, Bondarenko YV, Krat VB, Kushlinskii NE (2011). Neurospecific proteins in the serum of patients with brain tumors. Bull Exp Biol Med.

[36] Vos MJ, Postma TJ, Martens F, Uitdehaag BMJ, Blankenstein MA, Vandertop WP (2004). Serum levels of S-100B protein and neuron-specific enolase in glioma patients: a pilot study. Anticancer Res.

[37] Stupp R, Mason WP, van den Bent MJ, Weller M, Fisher B, Taphoorn MJB (2005). Radiotherapy plus concomitant and adjuvant temozolomide for glioblastoma. N Engl J Med.

[38] Wiesmann M, Missler U, Gottmann D, Gehring S (1998). Plasma S-100b protein concentration in healthy adults is age- and sex-independent. Clin Chem.

[39] Portela LVC, Tort ABL, Schaf DV, Ribeiro L, Nora DB, Walz R (2002). The serum S100B concentration is age dependent. Clin Chem.

[40] van Munster BC, Korevaar JC, Korse CM, Bonfrer JM, Zwinderman AH, de Rooij SE (2010). Serum S100B in elderly patients with and without delirium. Int J Geriatr Psychiatry.

[41] Wen PY, Macdonald DR, Reardon DA, Cloughesy TF, Sorensen AG, Galanis E (2010). Updated response assessment criteria for high-grade gliomas: response assessment in neuro-oncology working group. J Clin Oncol.

[42] Brommeland T, Rosengren L, Fridlund S, Hennig R, Isaksen V (2007). Serum levels of glial fibrillary acidic protein correlate to tumour volume of high-grade gliomas. Acta Neurol Scand.

[43] Ilhan-Mutlu A, Wagner L, Widhalm G, Wohrer A, Bartsch S, Czech T (2013). Exploratory investigation of eight circulating plasma markers in brain tumor patients. Neurosurg Rev.

[44] Rajendra A, Spinella PC, Drott HR, Dominguez TE, Sutton L, Helfaer M (2004). S-100beta protein—serum levels in children with brain neoplasms and its potential as a tumor marker. J Neurooncol.

[45] Kanner AA, Marchi N, Fazio V, Mayberg MR, Koltz MT, Siomin V (2003). Serum S100beta: a noninvasive marker of blood–brain barrier function and brain lesions. Cancer.

[46] Marchi N, Cavaglia M, Fazio V, Bhudia S, Hallene K, Janigro D (2004). Peripheral markers of blood–brain barrier damage. Clin Chim Acta.

[47] Syeda T, Muhammad Hashim AS, Rizvi HA, Hadi SM (2013). Serum S100B in patients with brain tumours undergoing craniotomy. J Coll Physicians Surg Pak.

